# Pharmacokinetics of Tylvalosin in Broiler Turkeys (*Meleagris Gallopavo*) After Single Intravenous and Oral Administration

**DOI:** 10.3389/fvets.2019.00355

**Published:** 2019-10-17

**Authors:** Mohamed Elbadawy, Mohamed Aboubakr, Amira Abugomaa

**Affiliations:** ^1^Department of Pharmacology, Faculty of Veterinary Medicine, Benha University, Moshtohor, Egypt; ^2^Faculty of Veterinary Medicine, Mansoura University, Mansoura, Egypt

**Keywords:** bioavailability, macrolides, pharmacokinetics, tylvalosin, broilers, turkeys

## Abstract

Pharmacokinetics of tylvalosin (TVN) were determined in eight broiler turkeys following a single intravenous (IV) and peroral (PO) administration of 25 mg/kg b.w using a crossover design with a 3 weeks washout period. Blood samples were taken between 0.083 and 24 h following TVN administration, plasma was separated and assayed for TVN concentrations by HPLC. The non-compartmental analysis was used to analyze plasma concentration-time curves. After IV administration, the pharmacokinetic profile was best described by a two-compartment model. The mean distribution and elimination half-lives were 0.382 and 5.71 h, respectively. The distribution volume at steady state, total body clearance and mean residence time were 8.30 L/kg, 1.17 L/h, and 7.16 h, respectively. After administering orally, the mean absorption half-life and absorption time of TVN was 0.955 and 2.31 h, respectively. The peak plasma concentration was 1.08 μg/mL and achieved at 2.0 h post-administration and the bioavailability was 53.3%. The plasma protein binding percent was 13%. For a successful clinical efficacy of TVN in broiler turkeys, a dosage regimen of 25 mg/kg b.w, given orally each day is recommended to keep efficient plasma levels above the MIC for most susceptible microorganisms.

## Introduction

Bacterial infections can endanger the lives of human beings and livestock or cause serious economic losses; therefore, antibacterial intervention is a critical issue. However, due to the frequent use of classical antibiotics, developing resistant bacterial strains continues to be a constant medical problem. New antibacterial agents can solve such an issue.

Tylvalosin is a new, broad-spectrum, third-generation veterinary macrolide antibiotic with 16-membered lactone ring and obtained from tylosin by the change of 3-acetyl-40-isovaleryl group to be acetylisovaleryltylosin tartrate (1, 2). As a macrolide, TVN inhibits the synthesis of bacterial protein by irreversible binding to 50S ribosome subunit of susceptible bacteria. Tylvalosin possesses a wide range of biological activities and significant therapeutic uses (3, 4). Against *Mycoplasma* species, TVN is extremely effective *in vitro* (5), and also against some isolates of *Brachyspira pilosicoli, Brachyspira hyodysenteriae*, and some anaerobes like *Clostridium* and *Bacteroides* species (6). Tylvalosin is used in swine for treating porcine proliferative enteritis, swine enzootic pneumonia and swine dysentery (1), and in poultry to control respiratory (*Mycoplasma* Species and *Ornithobacterium rhinotracheale*) and enteric (*Clostridium perfringens*) bacterial infections (6, 7). Moreover, TVN is better than tylosin in the higher intracellular penetration and accumulation inside respiratory and gut epithelial cells as well as phagocytic cells (3). Furthermore, TVN was shown to exhibit anti-inflammatory like characteristics and alleviates acute lung damage (2). Such data could suggest a substantially improved effect of TVN vs. tylosin. These features make TVN an attractive and prospective alternative against violent susceptible bacteria in the veterinary field. The safety and efficacy of macrolide antibiotics could be interpreted using their pharmacokinetic and pharmacodynamic data especially the cumulative time that the concentration exceeds the MIC (%T > MIC) for the time-dependent macrolides and AUC_24h_/MIC for the concentration-dependent ones, as azithromycin. However, there are few available data on avian pharmacotherapy and the shortage of pharmacokinetic data impedes the rational use of TVN in targeted avian species. Therefore, the current study was performed to characterize the disposition profile of TVN in broiler turkeys after single oral and intravenous administration.

## Materials and Methods

### Drug and Chemicals

Tylvalosin (Aivlosin®) was obtained as 62.5% water-soluble white granules (ECO Animal Health, London, UK). Each gram powder contains 625 mg of TVN as TVN tartrate. The internal standard, roxithromycin was obtained from Sigma-Aldrich Corp. (St. Louis, U.S.A.). Other chemicals and reagents consumed in the current study were acquired commercially and of HPLC grade.

### Experimental Turkeys

Eight clinically healthy broiler black turkeys (*Meleagris gallopavo*, 4 males and 4 females), weighing between 5.2 and 6.5 kg and of 11 weeks age were obtained from a local commercial turkeys farm and utilized to determine the pharmacokinetics of TVN. Turkeys were fed on a balanced ration free from drugs and water was supplemented *ad libitum*. Turkeys were housed in a hygienic room at 22 ± 1°C and 60 ± 10% humidity with a light cycle of 12 h/day for 2 weeks before being used to acclimatize the environment and for ensuring complete clearance of any antibacterial agents. All turkeys were clinically healthy before drug administration. The experimental protocol was approved ethically by the local Ethical Committee of the Faculty of Veterinary Medicine, Benha University, Egypt.

### Drug Administration and Blood Sampling

Before TVN administration, each bird was weighed to determine its dose. A Crossover design with a 3 weeks washout interval between the two routes of administration was used. Turkeys were divided into two groups (*n* = 4) and TVN was given as a single dose at 25 mg/kg b.w. (according to the manufacturer instructions) orally (intra-crop using oral gavages) and intravenously into the right brachial vein. Using the left brachial vein, blood samples (1 mL each) were obtained before and 0.083, 0.167, 0.25, 0.5, 1, 2, 4, 6, 8, 12, and 24 h post-drug administration using Venflon IV cannula and centrifuged at 1,600 *g* for 10 min. Plasma was aspirated and stored at −20°C until analyzed.

### Analytical Method

Tylvalosin concentrations in turkey's plasma were assayed by HPLC as described before (8) with some modifications. Briefly, roxithromycin (as an internal standard) was mixed with every standard, quality control sample and plasma sample at a level of 1 μg/mL. The plasma samples were mixed with 400 μL of acetonitrile including formic acid (0.1%), vortexed for 10 s and centrifuged at 20,000 *g* for 10 min at 4°C. Subsequently, the supernatant was gathered and evaporated to dryness in a thermostatically controlled water-bath maintained at 35°C (Rotavapor® R-114, Shibata Company, Tokyo, Japan). The residue was reconstituted in 150 μL mobile phase and defatted with 400 μL hexane, and the aqueous layer was collected and filtered by a 0.45 μm HPLC filter (Chromatodisc®, Kurabo Biomedical Company, Osaka, Japan) and 50 μL of the filtrate were injected into the HPLC column.

The HPLC system (Shimadzu Corporation, Kyoto, Japan) composed of a UV detector (SPD-6A), an integrator (Chromatopac C-R7A plus), a pump (LC-10AD) and a loop injector (Model 7125). The mobile phase consists of acetonitrile and (0.15 M) ammonium acetate buffer (49:51, v/v) solution. The analytical separation was accomplished by using Agilent TC-C18 column (5 μm, 4.6 × 250 mm, Agilent Technologies, USA) at 25°C. The flow rate was adjusted at 1 mL per min and the wavelength of the detector was 289 nm.

The calibration was carried out by spiking of 20 μL of diluted TVN standard solutions ranging between 0.019 and 20 μg/mL into 500 μL of blank turkey's plasma and assayed as mentioned above. The result showed that standard calibration curves of TVN were linear (*r* = 0.995). The limit of detection (LOD) was 0.039 μg/mL while limit of quantitation (LOQ) was 0.1 μg/mL. The average plasma recovery rate of TVN was 87.2%. The intra- and inter-day CV values ranged from 4.28 to 4.92% and 4.86 to 5.42%, respectively (*n* = 5, 3 times, 3 days).

### The Extent of Plasma Protein Binding of TVN

The plasma protein binding capacity of TVN was estimated *in vitro* by the ultrafiltration method as described previously (9). Different standards of TVN concentrations (as in standard calibration curves of TVN) were prepared, spiked to blank turkey's blank plasma samples in a triplicate manner for each concentration and vortexed for 20 s. Subsequently, samples were kept for 30 min at 37°C to allow binding between plasma protein and TVN. Thereafter, 1 mL of the sample was loaded into the sample reservoir of Ultrafree® centrifugal filter (UFC30LH00, a low-binding hydrophilic PTFE membrane, Millipore Corporation, Japan) with a pore diameter of 0.45 μm and subjected to ultrafiltration by centrifugation at 2,500 *g* at 37°C for up to 30 min or until the required volume of ultrafiltrate was obtained. The ultrafiltrate was assayed for TVN concentration as mentioned before. The extent of plasma protein binding was estimated with reference to the initial sample concentration according to following Equation;

(1) $$\begin{array}{l} \text{Plasma protein binding}\left(\%\right)\\ =\text{ ​}\left[\text{​}\frac{\text{spiked concentration }-\text{ ultrafiltrate concentration}}{\text{spiked concentration}}\text{​}\right]\times 100\;\;\;\;\;\;\; \end{array}$$

### Assay of TVN Pharmacokinetic Profile

Following IV injection, the plasma concentration vs. time curves of TVN fit well with the two-compartment model, while after PO administration it fit well with the one-compartment model. Thus, the curves recorded post IV [CP_IV_ (*t*)] and PO administration [CP_PO_ (*t*)] were characterized by Equations (2) and (3), respectively.

(2) $$\begin{array}{l} \text{C}{\text{P}}_{\text{IV}}\;\left(t\right)=\frac{\text{Dose}}{\text{V}}\;\left\{\frac{\propto -{\text{k}}_{21}}{\alpha -\beta }\cdot {\text{e}}^{-\alpha \cdot \text{t}}+\frac{{\text{k}}_{21-\beta }}{\alpha -\beta }\cdot {\text{e}}^{-\beta \cdot \text{t}}\right\} \end{array}$$

(3) $$\begin{array}{l} \text{C}{\text{P}}_{\text{PO}}\;\left(t\right)=\;\frac{\text{Dose}\cdot \text{F}\cdot ka}{\text{V}}\;\left\{\begin{array}{c} \frac{{\text{k}}_{21-\alpha }}{\left(\text{ka}-\propto \right)\left(\beta -\alpha \right)}\;\cdot {\text{e}}^{-\;\alpha \cdot \text{t}}\\ +\;\frac{{\text{k}}_{21}-\beta }{\left(\text{ka}-\beta \right)\left(\alpha -\beta \right)}\;\cdot {\text{e}}^{-\beta \cdot \text{ t}}\\ +{\frac{{\text{k}}_{21}-\text{ka}}{\left(\alpha -\text{ka}\right)\left(\beta -\text{ka}\right)}}_{\;}\cdot {\text{e}}^{-\text{ka}\cdot \text{t}} \end{array}\right\} \end{array}$$

Equations (2) and (3) were simultaneously fit (10–12) to the plasma concentration vs. time curve of TVN after IV and PO administration into the same turkey, respectively, to determine pharmacokinetic variables by the non-linear least square way using MULTI, a curve fitting program (13).

Several parameters have been calculated using a non-compartmental method of analysis (14). The AUC and AUMC were calculated by the trapezoidal method. The terminal elimination rate constant was estimated using four data points in the terminal part of the concentration vs. time curve by using the non-linear least-square iterative approach. The elimination half-life (t_1/2β_) was calculated as t_1/2β_ = 0.693/β, where β is the elimination rate constant. MRT = AUMC/AUC and Cl_tot_ = Dose/AUC_0−∞_. The absolute oral bioavailability (F) = AUC_PO_/AUC_IV_ × 100 and MAT = MRT_PO_-MRT_IV_. Distribution volume at a steady state (V_dss_) = Cl_t_/MRT_IV_.

## Results

**Figure 1:**
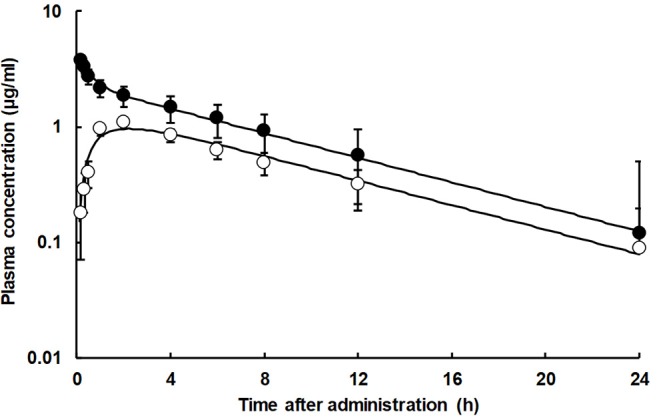
Semi-Logarithmic plot showing the plasma concentration vs. time curve of TVN in broiler turkeys following a single dose of 25 mg/kg b.w. administered intravenously (•) and orally (◦). Each mean ± SE (*n* = 8) are represented by each point and vertical bar, respectively. The IV and PO theoretical lines were depicted by Equations (2) or (3), respectively, using pharmacokinetic parameters in Table 1.

The mean plasma concentration vs. time profile of TVN following a single IV and PO administration of 25 mg/kg b.w. to broiler turkeys were graphed in Figure 1 and there was a good fitting between the observed points and theoretical curves. The pharmacokinetics data (Mean ± SE) estimated from the curve fitting and non-compartmental analysis were shown in Table 1. Following the IV injection, TVN concentration was sloped in a biphasic manner with a rapid and wide distribution and a long elimination half-life. After PO giving, TVN was quickly absorbed followed by slow elimination. The C_max_ was 1.08 μg/mL, reached (Tmax) at 2.0 h. The oral bioavailability of TVN was 53.3%. The *in vitro* plasma protein binding tendency of TVN was low (13 ± 0.785%).

**Table 1:** Mean (±SD, *n* = 8) plasma pharmacokinetic variables of tylvalosin in broiler turkeys determined following a single dose of 25 mg/kg b.w. administered intravenously (IV) and orally (PO).

**Parameter**	**IV**	**PO**
k_a_ (h^−1^)	—	0.745 ± 0.146
C_max_ (μg/mL)	—	1.08 ± 0.117
T_max_ (h)	—	2.00 ± 0.00
CP_0_ (μg/mL)	4.161± 0.688	—
α (h^−1^)	1.87± 0.393	—
β (h^−1^)	0.122± 0.011	—
t_1/2ka_ (h)	—	0.955 ± 0.148
t_1/2α_ (h)	0.382± 0.0826	—
t_1/2β_ (h)	5.71± 0.530	—
AUC (μg·h/mL)	22.1± 4.19	11.7 ± 2.95
AUMC (μg·h/mL)	159.4± 36.9	112.8 ± 38.9
Cl_t_ (L/h/kg)	1.17± 0.232	—
F (%)	—	53.3 ± 10.8
MRT (h)	7.16± 0.398	9.47 ± 1.06
V_dss_ (L/kg)	8.30± 1.23	—
MAT (h)	—	2.31 ± 0.83

*k_a_, absorption rate constant; C_max_, maximum plasma level; T_max_, time to achieve maximum plasma level; CP_0_, plasma concentration at time zero after IV injection calculated from dose/Vd; α and β, rate constants representing the slope of distribution phase and elimination phase, respectively; t_1/2_ka, t_1/2α_, and t_1/2β_, half-lives of absorption phase, distribution phase and elimination phase, respectively; AUC, area under the plasma concentration vs. time curve; AUMC, area under moment curve; Cl_t_, total clearance; F, fraction of drug absorbed systemically after oral administration; MRT, mean residence time; V_dss_, steady-state volume of distribution; MAT, meantime of absorption*.

## Discussion

Disposition of 16-membered lactone ring macrolides has been studied in a lot of avian species, and inter-species variations have been demonstrated as for tylosin in pigeons, quail, emus, and cranes (15) and chickens (16–18) as well as for tilmicosin in chicken (19) and turkeys (20). Tylvalosin itself in chickens during a pilot study showed different absorption profiles in between and within individuals when used on separate occasions (5). These differences in the disposition of macrolides among avian species require thorough pre-clinical assessment.

After IV injection of TVN in turkeys, the plasma concentration vs. time curve was tilted in a biphasic pattern, indicating that the disposition profile of TVN obeyed a two-compartment model. Similar data were recently recorded for TVN in turkey (21) and broiler chickens (22–24) and also for tylosin (18, 25, 26) and clarithromycin (27) in broiler chickens. Tylvalosin has good distribution profile in turkey as evidenced by a short (0.382 h) distribution half-life, probably due to extensive tissues distribution. Similarly, a short (0.076 h) t_1/2α_ of TVN in turkeys was recorded (21). Tylvalosin also showed a shorter t_1/2α_ (0.153 h) in broiler chickens at the same dose level (24) and after 10 mg/kg b.w. in laying hens [0.12 h (22)]. Similarly to TVN, the t_1/2α_ of tylosin tartrate (a chemically similar macrolide with time-dependent property) in broiler chickens after IV injection of 50 mg/kg b.w. was 0.385 h (25). Contrarily, shorter distribution half-lives were recorded for tylosin phosphate (0.07 h) and tylosin tartrate (0.09 h) in chickens but after IV administration of 10 mg/kg b.w. (18). In the present study, V_dss_ for TVN was 8.30 L/kg proposing a wide distribution of the drug in tissues of turkeys following IV injection. Similarly, the V_dss_ of TVN in broiler chickens was large (8.74 L/kg) after IV administration of the same dose (24). These are greater than those for tylosin reported earlier in broiler chickens as it was 0.69 L/kg (16), 0.94–1.09 L/kg (18), 5.30 L/kg (25), and 6.73 L/kg (26) after IV administration. This pharmacokinetic behavior is not surprising since macrolides are weak basic and highly lipophilic drugs with pKa values from 7.1 to 8.9 (pKa of TVN is 7.6) and low plasma protein binding tendency, thus these antibiotics move easily by non-ionic passive diffusion into tissues especially with a lower pH than blood (20, 21, 28). In the current study, the *in vitro* plasma protein binding tendency of TVN was low (13%) as is the case with other macrolides (18–30%) in most species (29). Similarly, tylosin (pKa 7.1) has a low ionization degree and a low binding to serum proteins (40%) is distributed widely in the body and attains greater tissue concentration than plasma levels (30). Our results showed a long (5.71 h) elimination half-life (t_1/2β_) of TVN in broiler turkeys after IV administration which is nearly similar to that of broiler chickens [6.67 h, (24)]. Contrarily, TVN showed short t_1/2β_ (0.788 and 0.61 h) in turkeys and laying hens after IV administration (21) and (22), respectively. The t_1/2β_ of tylosin in broiler chickens was also long [5.62 h (26) and 7.29 h (27)]. Contrarily, a short t_1/2β_ of tylosin [0.52 h (16) and 1.04–1.16 h (18)] was recorded in broiler chickens following IV injection of 10 mg/kg b.w. The total clearance of TVN was (1.17 L/h/kg) indicating a relatively quicker clearance in broiler turkeys. Nearly equal clearance values (1.498 and 0.953 L/h/kg) has been detected for TVN in turkeys and broiler chickens after IV injection (21) and (24), respectively. A larger value of TVN clearance (4.37 L/h/kg) was recorded in laying hens following IV injection of 10 mg/kg b.w. (22). In broiler chickens, higher clearance values after IV injection of 10 mg/kg b.w. of tylosin were 1.71 and 1.61 L/h/kg (18) and 5.3 L/h/kg (16).

Following PO administration, TVN showed rapid absorption from the alimentary tract of turkeys as indicated by short t_1/2ka_ (0.955 h) and MAT (2.31 h) as well as small ka (0.745/h). Similarly, rapid absorption of TVN was recorded where the t_1/2ka_ and ka were 0.875 h, 0.799/h (24), 0.94 h, and 0.69/h (23) in broiler chickens and the t_1/2ka_ was 0.74 h in laying hens (22). Moreover, shorter t_1/2ka_ values of TVN after PO dosing of 20 mg/kg b.w. using a rigid (0.471 h) or a flexible (0.175 h) catheter were reported also in broiler chickens (31). Also, the rapid absorption of TVN tartrate after PO giving to broiler chicken was recorded (6). Shorter t_1/2ka_ of tylosin tartrate [0.48 h, (18)], [0.19 h (25) and 0.3 h (26)] were also recorded in broiler chickens. The maximum plasma level (C_max_) of TVN after PO dosing to turkeys were 1.53 μg/mL and attained shortly (2 h) after administration. Additionally, the plasma concentration of TVN in the current study remained for 24 h higher than the MICs for TVN against several isolates of *Mycoplasma gallisepticum* (0.015–0.03 μg/mL) and *Mycoplasma synoviae* (0.015 μg/mL) isolated recently (Jan., 2017–Dec., 2018) from turkeys in Egypt (32) and TVN was found to be more effective than tilmicosin and tylosin in this study. Also, in another recent study (2014–2016), the MICs of TVN against 17 strains of *Mycoplasma synoviae* isolated from broiler turkeys originating from Central and Eastern Europe were ≤0.25 ug/mL (33). Nearly similar and different values of C_max_ and T_max_ of TVN in turkey and broiler chickens were recorded. In turkeys, the C_max_ and T_max_ of TVN were 0.637 μg/mL and 1.293 h (21). The C_max_ of TVN in broiler chickens were 2.11 and 1.23 μg/mL, attained at 2.03 and 1.72 h (23) and (24), respectively, and that of laying hens (20 mg/kg b.w.) was 22 μg/mL achieved at 0.86 h, respectively (22). Moreover, Cerda' et al. found in broiler chickens that the C_max_ of TVN (20 mg/kg b.w.) using a rigid or a flexible catheter were 6.104 and 1.641 μg/mL and achieved at 1.202 and 0.571 h, respectively (31). The C_max_ of tylosin in broiler chickens was 1.2 (0.18 and 0.44), 3.4 and 4.85 μg/mL attained at 1.5 (1.31 and 1.33), 1.08 and 1.32 h (16, 18, 25, 26), respectively. Our data showed that, the oral bioavailability of TVN was moderate (53.3%), higher than that of TVN in turkey [33.84% (21)] and nearly similar to that of TVN in chickens which was 60.26, 48.39, and 63.83% (22, 24, 34), respectively. However, in broiler chickens, the oral bioavailability of tylosin was from 35.4 to 40.6% (17), 13.74 to 27.0% (18), and 90.3% (25) and 89.2% (26). The differences in kinetic parameters between TVN and tylosin might be due to the differences in the chemical structure of both drugs (3). For anticipating the clinical efficacy of time-dependent antibacterial drugs, using the surrogate marker of the time free drug concentration in plasma is above the minimum inhibitory concentration fT ≥ /MIC (35), TVN would be a successful agent in turkeys for microorganisms with MIC ≤ 0.015–0.03 μg/mL after PO administration. Oral administration of 25 mg/kg b.w. of TVN every 24 h in broiler turkeys would be effective against several bacterial infections as chronic respiratory diseases caused by *Mycoplasma gallisepticum* and *Mycoplasma synoviae*.

In conclusion, administration of TVN (25 mg/kg b.w. each 24 h) might be highly effective for susceptible bacterial diseases in turkeys. However, further studies on tissue residues are necessary.

## Data Availability Statement

All datasets generated for this study are included in the manuscript/supplementary files.

## Ethics Statement

The experimental protocol was approved ethically by the local Ethical Committee of the Faculty of Veterinary Medicine, Benha University, Egypt.

## Author Contributions

ME contributed to the idea, design, performing the experiment, and writing the manuscript. MA contributed to pharmacokinetic analysis and revising the manuscript. AA performed the calculations, English check and grammars, and validation.

### Conflict of Interest

The authors declare that the research was conducted in the absence of any commercial or financial relationships that could be construed as a potential conflict of interest.
